# Computational Identification of Protein Methylation Sites through Bi-Profile Bayes Feature Extraction

**DOI:** 10.1371/journal.pone.0004920

**Published:** 2009-03-17

**Authors:** Jianlin Shao, Dong Xu, Sau-Na Tsai, Yifei Wang, Sai-Ming Ngai

**Affiliations:** 1 Department of Biology, The Chinese University of Hong Kong, Hong Kong, China; 2 Institute of Plant Molecular Biology and Agricultural Biotechnology, The Chinese University of Hong Kong, Hong Kong, China; 3 Department of Mathematics & Scientific Computing Key Laboratory of Shanghai Universities, Shanghai Normal University, Shanghai, China; 4 Department of Mathematics, Shanghai University, Shanghai, China; Michigan State University, United States of America

## Abstract

Protein methylation is one type of reversible post-translational modifications (PTMs), which plays vital roles in many cellular processes such as transcription activity, DNA repair. Experimental identification of methylation sites on proteins without prior knowledge is costly and time-consuming. *In silico* prediction of methylation sites might not only provide researches with information on the candidate sites for further determination, but also facilitate to perform downstream characterizations and site-specific investigations. In the present study, a novel approach based on Bi-profile Bayes feature extraction combined with support vector machines (SVMs) was employed to develop the model for Prediction of Protein Methylation Sites (BPB-PPMS) from primary sequence. Methylation can occur at many residues including arginine, lysine, histidine, glutamine, and proline. For the present, BPB-PPMS is only designed to predict the methylation status for lysine and arginine residues on polypeptides due to the absence of enough experimentally verified data to build and train prediction models for other residues. The performance of BPB-PPMS is measured with a sensitivity of 74.71%, a specificity of 94.32% and an accuracy of 87.98% for arginine as well as a sensitivity of 70.05%, a specificity of 77.08% and an accuracy of 75.51% for lysine in 5-fold cross validation experiments. Results obtained from cross-validation experiments and test on independent data sets suggest that BPB-PPMS presented here might facilitate the identification and annotation of protein methylation. Besides, BPB-PPMS can be extended to build predictors for other types of PTM sites with ease. For public access, BPB-PPMS is available at http://www.bioinfo.bio.cuhk.edu.hk/bpbppms.

## Introduction

Many proteins experience post-translational modifications through which they present structural as well as functional diversity and play important roles in many biological processes. Experimental identification and characterization of PTMs is labor-intensive and expensive in the absence of prior knowledge concerning PTMs. Computational prediction of PTM sites may provide researchers with information on the candidate PTM sites for further determination and downstream experimental characterizations.

Recently, protein methylation has attracted more and more attentions with the identification of an growing number of methyltransferases such as protein arginine methyltransferases (PRMTs) [1]–[3], histone lysine methyltransferases (HKMTs)[4]–[6]. Two previous works were done for protein methylation site prediction. Daily *et al* [7] built a predictor for arginine and lysine methylation using SVMs based on the hypothesis that PTMs preferentially occurs intrinsically disordered regions. They collected positive training datasets (methylated sites) from SWISS-PROT database (release 45)[8] and negative training datasets (non-methylated sites) from the same proteins, which include all arginines and lysines not marked as methylated. Examples in training datasets were encoded by a set of features including amino acid frequencies, aromatic content, flexibility scalar, net charge, hydrophobic moment, beta entropy, disorder information as well as PSI-BLAST profiles. In another team, Chen *et al.* [9] constructed the first online server MeMo for arginine and lysine methylation prediction via SVMs strategy. Positive training datasets are composed of peptides including the experimentally verified methylated lysines and arginines from SWISS-PROT database (release 48) plus manually curated data from PubMed literatures. Negative training datasets were collected through the similar way described in previous works [7], [10]. Examples in training datasets were represented by orthogonal binary coding scheme.

In the present study, a novel approach called Bi-profile Bayes was theoretically developed to extract features from training datasets, through which we constructed an online protein methylation prediction tool BPB-PPMS based on SVMs algorithm. As for encoding schemes (feature extraction approaches) employed in works [7], [9], each target site was represented in a single feature space manner (such as either intrinsically disordered regions for methylated peptide sequence or ordered regions for un-methylated peptide sequence) or through fixed binary coding scheme (fixed coding of each residue at any position for both methylated peptide sequence and un-methylated peptide sequence). Theoretically, each peptide sequence should exhibit different features in positive and negative feature spaces, respectively. It would be more informative to combine peptide sequence features in positive and negative feature spaces than single feature space or fixed binary encoding scheme. Bi-profile Bayes defines positive (methylated) and negative(un-methylated) feature spaces based on known experimentally verified data sets and each target site was represented in a bi-feature space manner, which was encoded by positive and negative feature vectors (see details in Methods). Results obtained from cross-validation experiments and test on independent data sets indicate the effectiveness of Bi-profile Bayes. BPB-PPMS is a novel general arginine and lysine methylation online tool and can provide probability information for prediction results other than that provided by MeMo.

## Methods

### Data collection

Methylated sites and non-methylated sites were collected as positive training datasets and negative training datasets, respectively. The sliding window strategy was utilized to extract positive and negative data from protein sequences as training data, which were represented by peptide sequences with arginine and lysine symmetrically surrounded by flanking residues. The positive training dataset are composed of all the arginines and lysines which were annotated as experimentally verified methylation on proteins from SWISS-PROT database (release 56.1). The negative training datasets include all the arginines and lysines that were not marked by any methylation information on the same proteins, the rational of which is that the resulting negative training samples are more likely to be non-methylation sites than those obtained by random as these proteins were experimentally investigated.

Candidate proteins for positive training datasets extraction were retrieved by searching information containing “Omega-N-methylated arginine”, “symmetric dimethylarginine”, “Omega-N-methylarginine” and “asymmetric dimethylarginine” for methylated arginines as well as “N6,N6,N6-trimethyllysine”, “N6,N6-dimethyllysine”, “N6-mehtylated lysine” and “N6-methyllysine” for methylated lysines under the description field in the feature table of Swiss-prot database. Total 363 candidate proteins containing methylated arginines and 977 candidate proteins containing methylated lysines were collected, respectively. Then, experimentally verified methylated arginines and lysines were recorded for later positive training datasets extraction by excluding those annotated by “By similarity”, “Potential” or “Probable” in the description field. In total, this yielded a total of 434 peptide sequences containing validated methylated arginines and 550 peptide sequences containing validated methylated lysines with sliding window size 11(the optimal window size for both arginine and lysine is 11 after several trials of 5, 7, 9, 11, 13, 15, 17 and data information regarding other sliding window sizes not shown here), respectively. Negative training datasets (non-methylated sites) for arginine and lysine were collected from sequences which contain experimentally validated methylated sites and included all arginines and lysines which were not annotated by any methylation information as described in previous studies[7], [9], [10].

### Homology reduction and data refinement

Training datasets obtained through the way introduced in the data collection section may present the homology and redundancy to some extent, which will overestimate the performance of the prediction model. Therefore, homology reduction or redundancy elimination requires to be performed. The way of redundancy elimination or homology reduction theoretically depends on the form of input data during the process of training, which is either the entire sequence or the peptide sequence. The corresponding homology reduction should be either sequence-based or window-based. Otherwise, it will overestimate the performance of the prediction model as well. Therefore, window-based homology reduction was applied in our case. Homology reductions within positive and negative datasets were performed with similarity threshold 70% between any two peptide sequences. Thus, 216 positives and 1980 negatives for arginine as well as 188 positives and 2157 negatives for lysine were obtained, respectively.

The size of the refined, non-redundant negative datasets is much larger than that of positive training datasets, which will result in bias prediction in favor of negative data. Although many approaches can be exploited to solve the imbalanced machine learning issues, under-sampling used in previous works [9], [10] was employed to overcome the imbalance between positive and negative datasets with the optimal reduction of negative data to 3 times the number of positive data in present study after trials of different ratios, which retains the original distribution of negative examples in order to avoid loosing diversity information as possible. Thus, the final negative training datasets contain 648 peptide sequences for arginine and 564 peptide sequences for lysine with peptide sequence length 11. The resulting negative datasets and positive datasets were pooled as the final training datasets and randomly split into 5 subsets, which share approximately equal number of items for 5-fold cross-validation training.

### Bi-profile Bayes for feature extraction

Suppose that we have an unlabeled sample 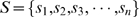 which denotes peptide sequence in our case, where each 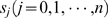 stands for one amino acid and 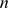 represents the length of peptide sequence, i.e. the size of sliding window in this study. 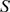 belongs to one of two categories 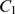 or 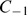, where 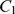 and 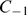 represent methylated sites (positive data) and non-methylated sites (negative data), respectively. According to Bayes' rule, the posterior probability of 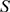 for these two categories can be given by
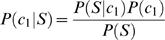
(1)

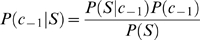
(2)where 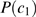 and 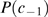 denote the prior probability for each category. Assume that 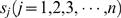 are mutually independent, Formula (1) and (2) can be rewritten as
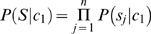
(3)

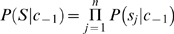
(4)By the above, Formulas (1) and (2) can be reformulated as
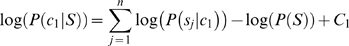
(5)

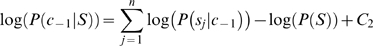
(6)where 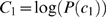 and 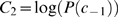. Thus, the decision function can be represented by formula (7)
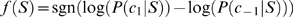
(7)Assume that prior distribution of category is uniform, namely, 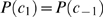, Formula (7) can be rewritten as
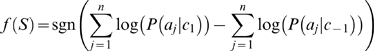
(8)Formula (8) can further be formulated as
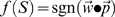
(9)where 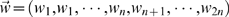 is weigh vector, 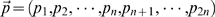 is the posterior probability vector. With respect to training sample 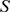, 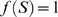 corresponds to class 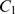 and 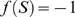 to class 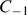. In this study, 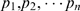 represents the posterior probability of each amino acid at each position in positive peptide sequence datasets (category 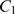) (positive feature space)and 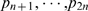 represents that in negative peptide sequence datasets (category 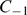) (negative feature space), which we call Bi-profile. The posterior probability can be estimated by the occurrence of each amino acid at each position in training datasets, which we define as position-specific profile.

### Profile generation and coding scheme

Two position-specific profiles for final model training, positive position-specific profiles and negative position-specific profiles, were generated through calculating the frequency of each amino acid at each position in the positive datasets and negative datasets, respectively. With respect to 5-fold cross-validation, position-specific profiles were produced based on the above-mentioned datasets minus the corresponding validation subset in each of five rounds of training in order to avoid overestimation of the performance. Through Bi-profile Bayes, each peptide sequence (positive or negative peptide sequence) can be represented and encoded by vector 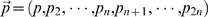, simultaneously containing positive and negative information, the dimension of which is two times that of sliding window.

### Support vector machines (SVMs) implementation and parameter optimization

In this contribution, prediction model was trained and built with LIBSVM package [11]. SVM is based on the structural risk minimization principle from statistical learning theory [12], which has been comprehensively applied to classification. With regard to binary classification, the SVM trains a classifier by mapping the input samples onto a high-dimensional space through kernel functions, and then seeking a separating hyperplane that differentiates the two classes with maximal margin and minimal error.

Radial basis kernel function 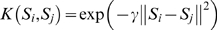 was selected for our SVM prediction system. Several preliminary trials were made on input window size for prediction model with 5, 7, 9, 11, 13, 15, 17 (sliding window size) amino acid peptide sequences centered by arginine and lysine. SVM parameter 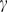 and penalty parameter 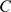 were optimized based on 5-fold cross-validation in a grid-based manner with respect to the above different length peptide sequences.

### Performance assessments

Accuracy (*Acc*), Specificity (*Sp*), Sensitivity (*Sn*), Receiver Operating Characteristic (*ROC*) curve, the area under *ROC* curve (*AUC*) and Matthews Correlation Coefficient (*MCC*) were utilized to assess the performance of prediction system. Acc denotes the percentage of both positive instances (methylated sites) and negative instances (non-methylated sites) correctly predicted. Sensitivity (true positive rate) and Specificity (true negative rate) represent the percentage of positive instances (methylated sites) correctly predicted and that of negative instances (non-methylated sites) correctly predicted, respectively. Due to the fact that calculation of *Sn* and *Sp* at a single threshold is potentially misleading, ROC cures is plotted to evaluate performance. A ROC curve is a plot of Sensitivity *versus* (1-Specificity) and generated by shifting the decision threshold. *AUC* gives a measure of classifier performance. An *AUC* of 1.0 indicates perfect classifier whereas an *AUC* of classifier no better than random is 0.5. The *MCC* is used in machine learning as a measure of the quality of binary classifications. It takes into account true and false positives and negatives and is generally regarded as a balanced measure which can be used even if the classes are of very different sizes. It returns a value between −1 and +1. A coefficient of +1 represents a perfect prediction, 0 an average random prediction and −1 the worst possible prediction. All of the above measurements were calculated in the case of 5-fold cross-validation and defined as follows:
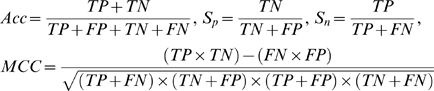
(10)where 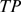, 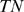, 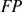 and 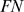 denotes the number of true positives, true negatives, false positives and false negatives, respectively.

## Results

### Performance of BPB-PPMS

**Figure 1:**
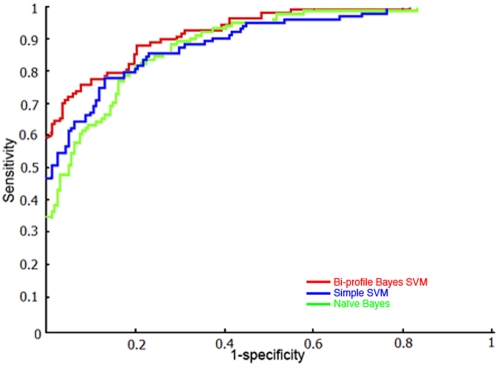
ROC curves to assess the prediction performance of three arginine prediction models. Red, blue, and green curve denotes 5-fold cross-validation prediction performance of Bi-profile Bayes SVM classifier, Simple SVM classifier and Naïve Bayes classifier, respectively. (The corresponding average AUC is 0.9254, 0.8958 and 0.8909, respectively.)

**Figure 2:**
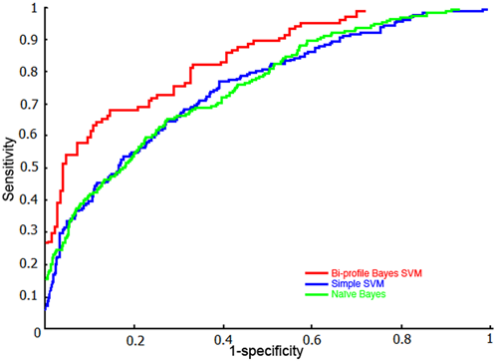
ROC curves to assess the prediction performance of lysine prediction model. Red, blue, and green curve denotes 5-fold cross-validation prediction performance of Bi-profile Bayes SVM classifier, Simple SVM classifier, Naïve Bayes classifier, respectively. (The corresponding average AUC is 0.8383, 0.7498 and 0.7581, respectively.)

The optimal parameters combination used for training model is shown in **Table 1**. All of the results were calculated based on the threshold value 0.5. The pooled datasets of positive training datasets and negative training datasets were randomly divided into five subsets with approximately equal number for cross-validation training. BPB-PPMS achieves the performance with a sensitivity of 74.71%, a specificity of 94.32% and an accuracy of 87.98% for arginine as well as a sensitivity of 70.05%, a specificity of 77.08% and an accuracy of 75.51% in the case of 5-fold cross-validation. To further evaluate the prediction performance, Receiver operating characteristic (ROC) [13] curves were plotted for the assessment of the performance of prediction models. The average AUC is 0.9254 for arginine and 0.8383 for lysine (Red curves in **Figure 1** and **Figure 2**), respectively.

**Table 1:** The optimal parameters and performance of BPB-PPMS.

Methylated residues	Optimal parameters				Performance				
	Sliding window size(a)	Type of Kernel	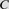 (c)	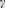 (d)	Sensitivity (%)	Specificity (%)	Accuracy (%)	AUC(e) (%)	MCC(f)
**Arginine**	11	RBF(b)	32	0.5	74.71	94.32	87.98	92.54	0.7729
**Lysine**	11	RBF	128	8	70.05	77.08	75.51	83.83	0.3400

The optimal parameter combination was determined in a grid-based manner introduced in LIBSVM packages[11].

(a) Here, input window size for SVMs is two times sliding window size.

(b) RBF, Radial Basis Function 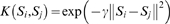.

(c)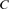, the penalty parameter of the error term in objective function.

(d)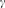, the parameter in Radial Basis Function.

(e) AUC, the area under ROC.

(f) MCC, Matthews Correlation Coefficient.

### Comparison with Naïve Bayes and simple SVMs classifiers

Typically, two strategies can be employed to perform standard comparison between distinct machine learning prediction models for binary classification problems, either through cross-validation experiments or test on the independent datasets given the same threshold value. It is logical for cross-validation performance comparison only when the training datasets for the prediction model is identical to each other. With respect to the independent test, the datasets employed should be not included into training datasets as well as no homologous to training datasets. As described in Methods section, the final training datasets of BPB-PPMS is not identical to previous works [7], [9]. Therefore, cross-validation performance comparison between BPB-PPMS and previous works [7], [9] under the uniform framework is infeasible and meaningless. Therefore, in order to evaluate the BPB-PPMS in the case of cross-validation circumstance, both Naïve Bayes classifier [14] and simple SVMs classifier s[11] without Bi-profile Bayes feature extraction were developed to identify potential protein methylation sites on the same training datasets as that of BPB-PPMS. Naïve Bayes classifier calculates the probability that a given example belongs to a certain class, which is based on the assumption that the features representing the example are conditionally independent given the class. Given an example *S*, described by its feature vector 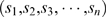, we are looking for a class *C* that maximizes the likelihood 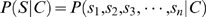. The assumption of conditional independence among the features, given the class, allows us to express this conditional probability 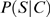 as a product of probabilities 
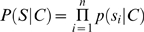
. Naïve Bayes classifier was trained using the input features produced from positive position-specific profile for positive training examples and negative position-specific profile for negative training examples (see Methods section for further details) through single feature space coding scheme, which was implemented via the package downloaded from http://fuzzy.cs.uni-magdeburg.de/~borgelt/bayes.html.

The principle of simple SVMs was briefly described in Methods section. simple SVMs classifier was trained through binary encoding for training samples and built with RBF kernel in LIBSVM package[11]. Both classifiers were evaluated via the same 5-fold cross-validation procedure as BPB-PPMS. All the performances were assessed under the circumstance of the same threshold value 0.5 and summarized in **Table 2**. The ROC curves for the assessment of the performance of three classifiers were plotted in **Figure 1** and **Figure 2**. The value of AUC is larger, the performance of model is better. As shown in **Figure 1**, red, blue, and green curve denotes 5-fold cross-validation prediction performance of Bi-profile Bayes SVM classifier, Simple SVM classifier and Naïve Bayes classifier for arginine methylation, respectively, the corresponding average AUC of which is 0.9254, 0.8958 and 0.8909, respectively. Likewise, red, blue, and green curve in **Figure 2** denotes 5-fold cross-validation prediction performance of Bi-profile Bayes SVM classifier, Simple SVM classifier, Naïve Bayes classifier for lysine methylation, respectively, the corresponding average AUC of which is 0.8383, 0.7498 and 0.7581, respectively. All the compared results obtained from **Table 2**, **Figure1**, and **Figure 2** suggest that PBK-PPMS outperformes Simple SVM classifier, Naïve Bayes classifier as well as Bi-profile encoding scheme is better than single feature space or binary coding scheme.

**Table 2:** Comparison among Naïve Bayes classifier, simple SVM classifier and BPB-PPMS classifier in the 5-fold cross-validation experiment on the same training datasets.

Methods	Methylated residues	Sensitivity (%)	Specificity (%)	Accuracy (%)	MCC
**Naïve Bayes**	Arginine	67.82	85.35	79.68	0.5379
	Lysine	66.31	73.19	71.62	0.2755
**simple SVM**	Arginine	70.11	89.01	82.90	0.6248
	Lysine	65.24	71.78	70.32	0.2502
**BPB-PPMS**	Arginine	74.71	92.46	86.80	0.7243
	Lysine	70.05	77.08	75.51	0.3400

### Comparison with previous works

Due to the absence of online server for the work done by Daily *et al* [7], efforts were just made to compare the performance between BPB-PPMS and MeMo. As mentioned in previous part, cross-validation performance comparison between BPB-PPMS and previous works [7], [9] under the uniform framework is infeasible and meaningless. With respect to independent dataset comparison, it's intractable to collect independent test datasets for both BPB-PPMS and MeMo since there is no any information regarding training datasets of MeMo for us. Therefore, unbiased comparison between BPB-PPMS and MeMo is infeasible as well. However, there is an attempt to further assess the performance of BPB-PPMS through test on the independent datasets, which were obtained by randomly choosing proteins with experimentally verified arginine and lysine methylation as well as non-homolog to those proteins used for training BPB-PPMS in PubMed literatures. The final random independent test datasets consist of 21 methylated lysines on 18 proteins as well as 12 methylated arginines on 11 proteins in the rat lumbar spinal cord[15] plus three methylated arginines which was most recently found in p53[16]. All of lysine methylation proteins and arginine methylation proteins were submitted to BPB-PPMS and MeMo. The performance based on the prediction results were summarized in **Table 3**. As shown in **Table 3**, the performance of MeMo is measured with a sensitivity of 20.00%, a specificity of 88.42% for arginine methylation proteins and a sensitivity of 9.52%, a specificity of 92.47% for lysine methylation proteins. BPB-PPMS achieves a sensitivity of 60.00%, with a specificity of 81.74% for arginine methylation proteins as well as a sensitivity of 71.43%, with a specificity of 91.51% for lysine methylation proteins at threshold value 0.5. Performance comparisons were performed at equivalent sensitivity or specificity value as a result of the absence of threshold choice on MeMo server. Therefore, attempt was made to adjust the threshold values of BPB-PPMS in order to obtain equivalent sensitivity or specificity value of MeMo. As for lysine methylation proteins, BPB-PPMS achieves a specificity of 98.65% compared to MeMo's 92.74% specificity at the identical sensitivity value 9.25% when the threshold value was set at 0.75. With respect to arginine methylation, BPB-PPMS is measured by a specificity of 88.56% and a sensitivity of 53.33% in the case of threshold value 0.80. By comparison, MeMo achives sensitivity value 20.00% at equivalent specificity of 88.42%.

**Table 3:** Performance of BPB-PPMS and MeMo on independent test datasets in terms of BPB-PPMS.

Server	Methylated residues	Threshold*	Sensitivity (%)	Specificity (%)	Accuracy (%)
**MeMo**	Arginine	-	20.00	88.42	87.22
	Lysine	-	9.52	92.47	91.11
**BPB-PPMS**	Arginine	0.5	60.00	81.74	81.36
		0.8	53.33	88.56	87.96
	Lysine	0.5	71.43	91.51	91.19
		0.75	9.52	98.65	97.19

* Prediction threshold value is not avalable in MeMo.

One important question was advanced whether comparison results shown in **Table 3** could suggest that BPB-PPMS would outperform MeMo since the independent test datasets is just in terms of BPB-PPMS. Theoretically, it is logical that the performance on datasets that are identical or homologous to training datasets should be better than that on independent datasets. Therefore, no matter whether independent datasets collected in present study is independent of training datasets of MeMo or not, it can be concluded that BPB-PPMS outperforms MeMo, at least at above equivalent sensitivity or specificity.

### Application of BPB-PPMS: a case study

Human immunodeficiency virus type 1 (HIV-1) Tat protein is a key player in HIV replication by virtue of its ability to dramatically increase gene transcription efficiency from the 5′ long terminal repeat (LTR) of the viral DNA[17]. The rate of transcription of the HIV-1 viral genome is mediated through the interaction of the viral protein Tat with the LTR and other transcriptional machinery [18]. Such specific interactions can be affected by the state of post-translational modifications on Tat. Tat protein is not included in our training datasets and can be employed for a case study

Recent studies[1], [19], [20] have shown that Tat can be specifically methylated by protein arginine methyltransferases 6 (PRMT6) on arginine residues at positions 52 and 53, resulting in a decreased interaction with TAR and cyclin T1 complex formation, therefore decreasing HIV-1 transcriptional activation. In order to map the region of Tat that is methylated by PRMT6, Boulanger *et al* [19] obtained three peptides that cover all the arginines of Tat. Tat peptide 1–14 contains arginine 7, peptide 49–63 contains the arginine-rich motif, and peptide 69–83 contains arginine 78. The findings obtained from *in vitro* methylation assays using these three Tat peptides demonstrate that Tat is methylated at region 49–63. Mutational analysis in another work done by Xie *et al* [1] was performed specifically on the 49-RKKRR-53 stretch, demonstrating that both R52 and R53 are targets for methylation. Our BPB-PPMS server predicts that arginine methylation of Tat can potentially occur at the site R7,R47,R52 and R53.

Most recently, Duyne *et al* [20] investigated the methylation of lysines on the peptide 45-ISYGRKKRRQ-54 of Tat. *In vitro* methylation assays show that lysine 50 and lysine 51 can be methylated by histone methyltransferases SETDB1, the SUV39-family of SET-domain containing proteins. They proposed that the methylation of Tat lysine 50 and 51 can result in a decrease in viral transcription. Our BPB-PPMS server predicts that there are two potentially methylated lysine sites K50, K51 on Tat with the probability 0.838290 and 0.8500, respectively. The prediction results regarding lysine methylation status on Tat are exactly in agreement with those obtained from experiments done by Duyne *et al* [20].

To verify whether these results to some extent reflect the generalization ability and robustness of BPB-PPMS, we checked the similarity among the positive training examples and peptide sequences including four experimentally verified methylated arginines and lysines. Interestingly, the maximum similarity with examples in positive training datasets is 40% for ISYGRK_(50)_ KRRQR, 50% for SYGRKK _(51)_ RRQRR, 70% for YGRKKR_(53)_ RQRRR and 70% for GRKKRR_(54)_ QRRRP, respectively. Therefore, it can be concluded that the results from Tat protein study, to some extent, verify the generalization ability of BPB-PPMS. The detailed prediction results on Tat protein through three classifiers are shown in **Table 4**.

**Table 4:** Potential methylation sites predicted on Tat protein (P04610) through BPB-PPMS, Simple SVMs, and Naïve Bayes classifiers.

Experimentally verified methylation sites on Tat protein	Potential methylation sites predicted on Tat protein		
	BPB-PPMS	Simple SVMs	Naïve Bayes
**K50,K51,R52,R53**	**K50(0.83829)**, **K51(0.8500)**, R7(0.992805), R49 (0.917059), **R52(0.991765)**, **R53(0.941735)**	K28(0.9012), **K50 (0.889106)**, K71(0.7622), **R53(0.78301)**	K19 (0.6577), **K50(0.9250)**, K71(0.8119233)

The numbers in bracket denote the predictive probability of methylation at corresponding sites.

## Discussion

In this work, a novel online tool (BPB-PPMS) was developed to predict arginine and lysine methylation sites from sequences using Bi-profile Bayes feature extraction combined with SVMs. Results from cross-validation experiments (**Table 2**) indicate that BPB-PPMS outperforms both Naïve Bayes and simple SVMs classifiers due to the fact that Bi-profile Bayes coding scheme possesses advantages over binary coding scheme and single feature space coding scheme. In addition, performance on independent datasets for BPB-PPMS and MeMo (**Table 3**) shows that BPB-PPMS outperforms MeMo, even though independent datasets is only in terms of BPB-PPMS, which might result from two factors. One is the more diverse training datasets employed in BPB-PPMS (training datasets collected up to Sep, 2008) than those used in MeMo (training datasets collected before submission of MeMo work, Jan-19, 2006). Another factor is that, as indicated by **Table 2**, Bi-profile Bayes coding scheme used in BPB-PPMS outperforms binary coding scheme utilized by MeMo.

Prediction models for functional sites can provide valuable information for future experimental designs. However, information regarding negative training/test datasets (definitely determined non-functional sites) is scarce, which is a choke point for the development of prediction models. Most of the existing tools (classifiers) for prediction of PTM sites from sequences[7], [9], [10], [21]were developed through various kinds of machine learning approaches using experimentally verified PTM sites and putative non-PTM sites as training datasets. The performance of classifiers not only depends on the robustness of machine learning approaches, but also whether the features extracted from training datasets accurately reflect those of PTMs or non-PTMs. Therefore, the quality of training datasets directly influences the classification boundary and subsequent prediction performance. The putative negative examples employed in most of prediction models are generated based on either features of known functional sites[14] or “accept or reject” rule[7], [9], [10]. A recent study[22] proposed maximum distance minimum redundancy approach to generate initial negative training datasets and predicted non-coding RNAs from unlabeled data, which may be an useful way for the generation of negative training examples with high confidence and could be extended to the investigation of PTM site prediction modeling. Although it is to some extent rational for the generation of putative negative training examples (non-PTM sites) from all of the remaining sites on proteins containing experimentally verified sites (methylated arginines and lysines in our case)[7], [9], [10], some putative negative examples are in fact false, which will contribute to false negative prediction. Therefore, as more validated methylated sites from high throughput proteomic experiments become available, it should be possible to further improve the reliability of predictions. In addition, the inclusion of structural information into modeling process could be another way to enhance the prediction performance since methylation is an enzymatic process and the interactions between methylated sites and enzymes concerned should be structurally satisfied.
